# The Differential Effects of Tai Chi vs. Brisk Walking on Cognitive Function Among Individuals Aged 60 and Greater

**DOI:** 10.3389/fnhum.2022.821261

**Published:** 2022-03-17

**Authors:** Ye Yu, Erfei Zuo, Scott Doig

**Affiliations:** ^1^School of Sports Science, Changsha Normal University, Changsha, China; ^2^School of Physical Education, Xiangnan University, Chenzhou, China; ^3^Department of Education and Kinesiology, Limestone University, Gaffney, SC, United States

**Keywords:** Tai Chi, exercise, cognition, memory, old people

## Abstract

**Purpose:**

The aim of this study was to investigate the differential effects of Tai Chi vs. brisk walking on cognitive function among individuals aged 60 and greater.

**Patients and Methods:**

For participant recruitment, a health talk was arranged at two communities in which two different exercise modalities (Tai Chi and brisk walking) were assigned to participants of each community free of charge. The intervention programs lasted 10 weeks, with three 60-min training sessions per week. General cognitive ability and specific cognitive outcomes were measured using the Chinese version of the Montreal Cognitive Assessment (MoCA).

**Results:**

A significant interaction on total scores of the MoCA was observed (*F* = 11.15, *p* < 0.05). *Post hoc* analysis indicated significant improvements on general cognitive performance as measured in performance on the MoCA for both exercise groups at the end of 10 weeks. A significant interaction was only observed on the delayed recall sub-domain (*F* = 12.93, *p* < 0.001). Results from *post hoc* analysis indicate that the Tai Chi group had a significantly better memory performance relative to brisk walking group (*p* < 0.05). Specifically, significant improvement was observed in Tai Chi group (*p* < 0.05), but not in the brisk walking group. Both exercise groups demonstrated significant improvements from baseline to Week 10, which emerged in visualspatial ability (*p* < 0.05) and attention performance (*p* < 0.001). Lastly, animal naming and orientation significantly benefited from brisk walking (*p* < 0.05) and Tai Chi training (*p* < 0.05), respectively.

**Conclusion:**

Tai Chi and brisk walking as the most commonly used, culture-specific mind-body exercise method have been proven to be effective in improving general cognitive performance and specific cognitive domains. Furthermore, differential effects of two different exercise modalities on cognitive domains were observed, which has provided insightful information for customized exercise programs. Finally, aging individuals who are experiencing cognitive decline should either take Tai Chi classes regularly or engage in brisk walking, which could contribute to brain health.

## Introduction

The number of individuals aged 60 years old and greater is significantly increasing, with data from the world Health Organization indicating 1 billion in 2019. The global proportion of this age group will contiguously elevate and reach 1.4 billion by 2030 (Miniscalco et al., 2006). To be consistent with the global trend in population aging, China–a developing country–is also undergoing a significant increase (Han et al., 2020). Data has indicated that individuals aged 60 years and over had reached roughly 250 million in 2018, accounting for about 18% of the total population (Chunli, 2019). This aging population is facing health challenges, particularly a widely recognized decline on cognitive function (refer to multiple mental skills including attentional ability, memory, language, executive function/visuospatial ability) (Zou et al., 2019a). Such age-related cognitive decline could generate substantial economic burdens and affect the personal life of an individual. Thus, looking for approaches to mitigate the progress of cognitive decline with normal aging is urgently needed.

Until now, pharmacological methods have been found to be ineffective at reversing age-related cognitive decline, with many reporting side effects following use of these drugs (Andrade and Radhakrishnan, 2009). Against this background, some researchers have started to shift their attention to modifiable lifestyle factors including smoking, alcohol use, and physical activity. Of them, physical activity has been observed as an effective approach to improve cognitive function of aging populations with healthy individuals or those with cognitive impairment (Demurtas et al., 2020). For example, multiple meta-analytical reviews conducted by several research groups indicated that exercise improved cognitive function among middle-aged and older individuals (Kelly et al., 2014; Northey et al., 2018; Sanders et al., 2019; Wu et al., 2019). Physical activity refers to musculoskeletal behaviors with energy expenditure. It contains a wide range of modalities such as aerobic exercise, resistance training, endurance training, high-interval intensity training. Under the umbrella of exercise, the superior effects on cognitive function were observed following mind-body exercises (Tai Chi, and Yoga) (Herold et al., 2018; Wu et al., 2019; Yu et al., 2021). Furthermore, Tai Chi as a unique exercise modality has recently gained increased global popularity, especially among older individuals, which may be attributed to the slow and gentle movements, deep breathing techniques, and sensorimotor training.

With the increasing number of experimental studies investigating the cognitive benefits of mind-body exercises among aging populations, several reviews were conducted, indicating that mind-body exercises have the potential to improve cognitive functioning in older individuals (Wu et al., 2013; Wang et al., 2018; Lim et al., 2019). Of note, the majority of these previous studies focused on single mind-body exercise vs. non-active control or the comparative effects of two different types of mind-body exercises (Zou et al., 2019b). Very few studies have investigated the differential effects of exercise type on cognitive function in elderly populations (Demurtas et al., 2020). A cross-sectional study was recently published (Yue et al., 2020), indicating that as compared to older adults who engaged in brisk walking for at least 5 years (60 min × 5 times per week) Tai Chi practitioners demonstrated significantly better behavioral measure/cognitive function (delayed recall-memory function) and greater hippocampus. Researchers concluded that such superior effects may be attributed to complexity of Tai Chi movements (motor-coordinative training and movement sequence) and mindfulness-based training. Given that results from a cross-sectional study cannot establish a cause-and-effect relationship, more experimental studies are needed to substantiate this promising finding. Thus, the aim of this study was to investigate the effects of Tai Chi vs. brisk walking on cognitive function among older individuals aged 60 and over. Results from this study would provide insightful information for health professionals to customize exercise training regiments among healthy older adults who suffer normal cognitive decline.

## Materials and Methods

### Study Participants and Group Assignment

In the present study, an experimental design was used to investigate the effects of Tai Chi vs. Brisk Walking on cognitive function among individuals aged 60 and greater. For participant recruitment, a health talk was arranged at two different communities in which only information about physical and physiological benefits of exercise was presented but not brain health (to minimize the social desirability). To be included in this study, individuals must meet the inclusion criteria: (a) healthy participants at the age of 60 or greater; (b) able to walk independently and perform daily living activities–which would allow participants to successfully learn Tai Chi forms; (c) baseline MoCA [the Montreal Cognitive Assessment Scale] scores should reach at least 26, which reflects normal cognitive level. Likewise, individuals who were interested in this study but belong to one of the following criteria were excluded: (a) being diagnosed with any major diseases like stroke, heart attack, chronic obstructive pulmonary disease, or diabetes mellitus; (b) hearing and/or vision impairments which would limit their ability to follow instruction during exercise training and assessment; (c) engagement in any behavioral or exercise training program in previous 2 months. Two different types of exercise modalities were arranged at two communities in which one group attended Tai Chi and another engaged in brisk walking. Study protocol (PN-2020-034) was approved by the Xiangnan University ethical committee.

### Exercise Training Modalities

Participants who volunteered to participate in Tai Chi training were informed about the intervention duration, frequency of outcome measures, and associated policies. Specifically, Tai Chi intervention duration lasted 10 weeks (60-min training session × 3 times per week) in which 24-style form was taught by two certified instructors (Zou et al., 2019b). Prior to each Tai Chi training session, an 8-min warm up was arranged including whole-body stretching and footwork training (the foundation for Tai Chi form practice). And then participants learned Tai Chi moves and memorized its associated movement sequences, followed by another 5-min cool-down section. To maximize the teaching effectiveness and quality, Tai Chi groups were separated into groups of 15 participants. To be consistent in terms of teaching, two instructors were given time to communicate with each other after their own Tai Chi training session. Participants in the brisk walking group underwent the same intervention duration in which they received 60-min walking sessions 3 times per week. All brisk walking sessions were instructed by a sophisticated instructor who engaged in outdoor sports for 20 years. Of note, the heart rate was collected in one-third of each group to monitor the exercise intensity; 65–75% of the maximum heart rate (220–age) was targeted zone.

### Outcome Assessment

Demographic data were collected in the present study, including age, gender, educational level, handedness, height, and weight (can be calculated for BMI). Of note, this information was self-reported. Global cognitive function and its associated subdomains were measured using MoCA.

The Chinese version of the Montreal Cognitive Assessment (MoCA) was used to measure global cognitive performance and its individual abilities (Lu et al., 2011), which was administered by trained research assistants. This assessment tool consisted of 30 items within the following domains: Orientation, delayed recall, visuospatial ability/executive functioning, language abilities, abstraction, animal naming, attention, and clock-drawing test. It took approximately 12–15 min to complete the entire assessment. Each participant could obtain a maximum of 30 points. Individual scores across domains ranged between 3 and 6: (a) to assess orientation [6 points], participants were asked to state the date, month, year, day, place, and city; (b) delayed recall [5 points] was assessed; (c) executive function/visuospatial ability [5 points] were measured *via* Trail Making B–participants were asked to draw a line to correctly connect alternating digits and numbers like 1-A and 2-B; (d) language abilities [3 points] required participants to repeat two sentences correctly and then list all of the words that could be recalled starting with the Letter F; (e) to measure abstract reasoning [2 points], participants were asked to explain how two items were alike [train and bicycle]; (f) the animal naming test [3 points] consisted of three pictures of animals presented to participants who were asked to name each item; (g) the attention task [6 points] was comprised of a series of numbers which participants were asked to recite forward and backward.

### Experimental Procedures

**FIGURE 1:**
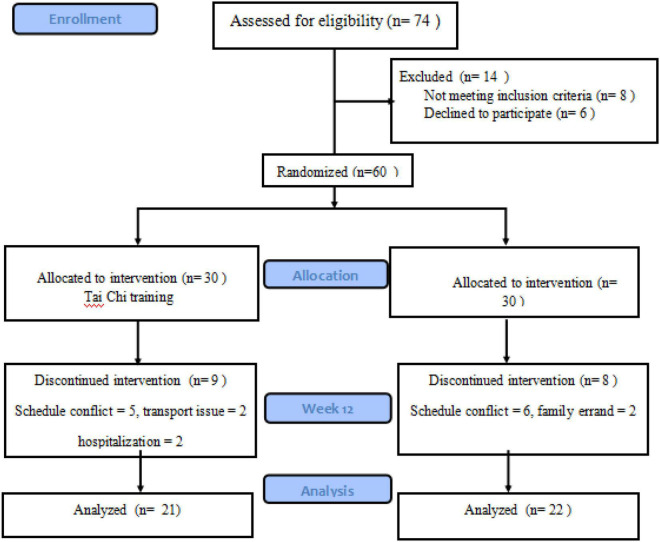
Flowchart of enrollment, baseline, and post-assessment.

A health talk was arranged at two different communities in which the majority of audiences were older adults aged sixty and greater. Immediately after this event, participants were invited to attend one of the exercise programs. Only those who met the eligibility requirements completed the baseline assessment. Eligible participants were informed that they could withdraw from the 10-week intervention exercise program at any time. Post-assessment was also performed after the 10-week intervention period within one week. Trained graduate students were responsible for cognitive assessments. Nine participants (5 males and 4 females) did not complete the 10-week Tai Chi intervention due to the following reasons: (a) schedule conflicts = 5 participants; (b) hospitalization = 2 participants; (c) transport issue = 2 participants. Similarly, participants in the Brisk Walking group dropped out because of: (a) schedule conflicts = 6 participants; (b) family errand = 2 participants. Procedures are detailed in Figure 1.

### Statistical Analysis

Demographic data, MoCA and its associated sub-domain scores were collected for baseline analyses. The number and percentage were used to present results of categorical variables while mean and standard deviation (SD) were reported for continuous variables. A chi-square test and independent *t*-test were used for categorical and continuous variables, respectively. A two-way ANOVA with repeated measure was conducted to investigate the effects of Tai Chi vs. brisk walking on cognitive function. *Post hoc* analysis was carried out using a Bonferroni test when significant main and group × time interaction effects were found. A *p* value of less than 0.05 was used to determine significance level.

### Patient and Public Involvement

Patients and/or the public were not involved in the design, or conduct, or reporting, or dissemination plans of this research.

## Results

The number of male and female participants in both exercise groups were relatively equal (*p* = 0.658). Specifically, there were 10 male participants in Tai Chi group, accounting for 47.6% of the total number, while 9 male participants (40.9%) engaged in brisk walking training. With respect to educational level, a relatively equal number of participants across three different educational levels were observed (*p* = 0.859). Participants in the Tai Chi group had a mean age of 64.90 ± 3.37 years old, while the brisk walking group was 66.86 ± 5.74; no significant group difference was observed on age. BMI of Tai Chi and brisk walking were 24.03and 23.23 kg/m2, respectively. No significant difference between Tai Chi (26.38 ± 1.35) and brisk walking (26.59 ± 1.40) groups was observed in baseline MoCA scores (*p* = 0.513). Participant descriptive statistics are detailed in Table 1.

**TABLE 1:** Demographic information.

Demographic	Tai Chi	Brisk walking	Statistic (df)	*p*-value
Gender			χ2(1) = 0.196	0.658
Male	10 (47.6%)	9 (40.9%)		
Female	11 (52.4%)	13 (59.1%)		
Educational Level			χ2(1) = 0.305	0.859
College or greater	5 (23.8%)	6 (27.3%)		
Associate degree	13 (61.9%)	14 (63.6%)		
High school or below	3 (14.3%)	2 (9.1%)		
Age (years)	64.90 ± 3.37	66.86 ± 5.74		0.183
BMI (kg/m2)	24.03 ± 1.48	23.23 ± 2.74		0.242
MoCA	26.38 ± 1.35	26.59 ± 1.40		0.513

*MoCA, Montreal cognitive assessment.*

A significant interaction on total scores of the MoCA was observed (*F* = 11.15, *p* < 0.05). Results from *post hoc* analysis indicate significant improvements on general cognitive performance as measured by the MoCA in both exercise groups for Week 10 of the intervention.

With respect to sub-domains, only a significant interaction on delayed recall was observed (*F* = 12.93, *p* < 0.001). Results from *post hoc* analysis indicate that Tai Chi group had significantly better memory performance relative to Brisk Walking group (*p* < 0.05). Specifically, significant improvement was only observed in Tai Chi group (*p* < 0.05), but not in the Brisk Walking group. Both exercise groups demonstrated significant improvements from Week 10 to baseline, which emerged in only visualspatial ability (*p* < 0.05) and attention performance (*p* < 0.001). Lastly, animal naming and orientation significantly benefited from Brisk Walking (*p* < 0.05) and Tai Chi training (*p* < 0.05), respectively. Subdomain scores are detailed in Table 2.

**TABLE 2:** Montreal cognitive assessment and its associated sub-domain scores.

Outcomes	Tai Chi		Brisk walking		*F*-value	
			
	Baseline	Week 1.0	Baseline	Week 10	Group	Group × Time
MoCA	26.38 ± 1.35	29.33 ± 0.73**	26.59 ± 1.40	28.32 ± 1.21**	3.48	11.15*
Visuospatial Ability	4.62 ± 0.67	4.90 ± 0.30*	4.55 ± 0.74	4.82 ± 0.39*	0.295	0.006
Animal Naming	2.86 ± 0.35	3.00 ± 0	2.73 ± 0.46	3.00 ± 0*	1.071	1.07
Attention	4.71 ± 0.78	5.86 ± 0.36**	4.64 ± 0.90	5.5 ± 0.67**	1.396	1.48
Language Ability	2.76 ± 0.54	2.90 ± 0.3	2.86 ± 0.35	2.91 ± 0.29	0.395	0.36
Abstraction	1.95 ± 0.22	2.0 ± 0	1.82 ± 0.39	1.82 ± 0.39	3.653	0.34
Delayed Recall	4.10 ± 0.83	4.81 ± 0.40*	4.77 ± 0.43	4.82 ± 0.39	6.26*	12.93**
Orientation	5.38 ± 0.50	5.86 ± 0.36*	5.23 ± 0.69	5.46 ± 0.67	3.53	1.81

MoCA, Montreal cognitive assessment; *, p < 0.05; **, p < 0.001.

## Discussion

In the present study, researchers have investigated the effects of two different exercise modalities on cognitive function among individuals aged 60 and greater. Specifically, this quasi-experimental study not only focused on general cognitive performance following the 10-week Tai Chi and Brisk Walking training, but researchers were also interested in its associated cognitive domains. Results of this study indicated that individuals who were at the age of 60 can receive cognitive benefits following both exercise modalities, with differential effects on specific cognitive domains. Potential mechanism of exercise-induced cognitive benefits will be discussed below.

As mentioned previously, age-related cognitive decline has been widely recognized, which typically deteriorates (converting to dementia) without any preventive strategies. To this end, researchers and health professionals have been searching for effective approaches to decelerate the progress of cognitive decline and improve brain health to lead to a better quality of life. When the etiology of cognitive decline remains largely unclear, associations with modifiable lifestyle behaviors and cognitive function for aging populations have attracted greater attention from the research community. Of these modifiable lifestyle factors, physical activity or exercise is of particular research interest. Specifically, researchers have investigated the cognitive benefits of acute and chronic exercise among aging populations. Accumulating evidence indicate the beneficial effects of aerobic exercise and resistance training for cognitive improvement and brain health. Recently, researchers have started to investigate the effects of mind-body exercise (Tai Chi, Yoga, and Baduanjin Qigong) on cognitive function in aging populations. Such exercise modality has gained increasing popularity globally, especially among aging populations–it is mainly attributed to slow pace movement features which older practitioners can follow. In addition, deep breathing practice could help to alleviate negative emotion and loneliness experienced by older individuals.

After the 10-week intervention period, Tai Chi and brisk walking training programs effectively improved visualspatial ability and attention ability. Such results of the present study are in line with previous studies (Muiñs and Ballesteros, 2018; Zou et al., 2019a; Nemoto et al., 2020). For example, Tai Chi has a greater emphasis on eye-hand-feet coordination in which participants were required to place their feet correctly and dynamically carry out weight shifting in order to maintain balance. Visualspatial ability and attention was consistently trained throughout the 10-week intervention period. Interestingly, Brisk Walking and Tai Chi training improved animal naming and orientation, respectively. Brisk Walking training was performed as a group in which participants had the opportunity to communicate, which possibly had positive effects on this outcome. As mentioned previously, Tai Chi form involved multi-directional movement, which resulted in better performance in the orientation task. With respect to delayed recall, Tai Chi group demonstrated better memory performance relative to brisk Walking group. Furthermore, the significant improvement was only observed in Tai Chi group, but not in the Brisk Walking group. Findings suggest that Tai Chi may have the superior effects on memory function, which is supported by a previous study (Zou et al., 2019a). Such results may be attributed to unique features which require participants to memorize movement sequences during Tai Chi training in comparison with brisk walking that only involved simple movement patterns. Unsurprisingly, improvements in multiple subdomains were observed, which in turn contributed to significantly greater general cognitive performance (as measured by the MoCA) at Week 10 relative to baseline.

Tai Chi training-induced cognitive function may be also attributed to molecular and cellular changes (first level) as well as functional and structural brain changes (second level) (Brown et al., 2013; Gomez-Pinilla and Hillman, 2013; Marmeleira, 2013; Norman et al., 2018). With respect to the first level, brain-derived neurotrophic factor (BDNF) and other neurotrophic factors have been increasingly recognized to associate with cognitive function. A significant number of animal and human studies indicated that regular exercisers with greater aerobic fitness was associated with basal levels of BDNF, which contributed to larger hippocampal volume and better memory function (Currie et al., 2009; Erickson et al., 2010, 2011). Specifically, a well-designed study investigated the effects of Tai Chi on cognition and associated plasma biomarkers among older adults with amnestic mild cognitive impairment (Sungkarat et al., 2018). After a 6-month intervention period, in comparison with a control group, Tai Chi group demonstrated significantly better memory performance and executive function, which was mediated by an upregulation of BDNF. Thus, 12-week Tai Chi training can improve aerobic fitness and elevate BDNF levels, which in turn facilitates memory performance enhancement. When taking a look at the second level, a leading study conducted by Erickson indicated that aerobic exercise can increase the size of the hippocampus and enhance memory performance following a 12-month intervention period (Erickson et al., 2011). In this study, a total of 120 older individuals were randomly assigned into either aerobic exercise (3 × 60 min per week) or stretching control; memory function and brain structure were measured across three time points. Interestingly, older participants in the aerobic exercise group demonstrated significantly increased size of the anterior hippocampus (2%), which effectively reverse this brain structure loss due to normal aging. Furthermore, this study reported greater size of hippocampus was linked to higher serum levels of BDNF. Such brain structure change and molecular change ultimately led to significant improvement in spatial memory performance. Collectively, Tai Chi training-induced cognitive benefit may be due to the BDNF and the increased size of hippocampus.

### Strengths and Limitations

Tai Chi as a mind-body exercise has been identified to improve cognitive function of aging population. Culture-specific Tai Chi and Brisk Walking are easy-to-learn in Chinese society, especially in aging populations who commonly used such exercises for health benefits. Investigation on the cognitive benefits of Tai Chi vs. Brisk Walking have rarely been done simultaneously. Thus, results of the present study have added value to the existing literature. Several limitations in this study should be admitted. Firstly, this is a non-randomized controlled trial in which participants in both groups may have high expectations. To further verify the positive effects of Tai Chi on cognitive function of the elderly, future studies on this similar topic should include at least a wait-list control group. Secondly, individuals aged 60 and greater were commonly reported with depression and loneliness that were not measured in this study. It remains unclear if exercise-induced effects could alleviate these negative emotions, leading to better cognitive performance. Thirdly, this study only included assessments of baseline and post-intervention. Further investigation is required to assess if such positive effects persisted past the end of the study. Fourth, blinding of assessors may also confound the results of the present study. Fifth, economical level of participants were not collected in this study, which requires attention in the future studies. Lastly, a non-active control should be added in future studies so that results on the beneficial effects of two exercise modalities are more persuasive.

## Conclusion

Tai Chi and brisk walking as the culture-specific mind-body exercise and the most commonly used method have been proven to be effective in improving general cognitive performance and specific cognitive domains. Furthermore, the differential effects of two different exercise modalities on cognitive domains are observed, which has provided insightful information for customized exercise programs. Finally, aging individuals who are experiencing cognitive decline should either take Tai Chi classes regularly or engage in brisk walking, which could contribute to brain health.

## Data Availability Statement

The original contributions presented in the study are included in the article/supplementary material, further inquiries can be directed to the corresponding author.

## Ethics Statement

The studies involving human participants were reviewed and approved by the Xiangnan University Ethics Committee. Informed consent was obtained from all subjects involved in the study prior to the starting of this study.

## Author Contributions

YY and EZ: conceptualization, validation, investigation, and data curation. YY and SD: methodology and formal analysis. YY: software and resources. YY, EZ, and SD: writing—original draft preparation and writing—review and editing. EZ: visualization, supervision, project administration, and funding acquisition. All authors have read and agreed to the published version of the manuscript.

## Conflict of Interest

The authors declare that the research was conducted in the absence of any commercial or financial relationships that could be construed as a potential conflict of interest.

## Publisher’s Note

All claims expressed in this article are solely those of the authors and do not necessarily represent those of their affiliated organizations, or those of the publisher, the editors and the reviewers. Any product that may be evaluated in this article, or claim that may be made by its manufacturer, is not guaranteed or endorsed by the publisher.
